# Stretch Sensor: Development of Biodegradable Film

**DOI:** 10.3390/s24020683

**Published:** 2024-01-21

**Authors:** Uldis Žaimis, Jūratė Jolanta Petronienė, Andrius Dzedzickis, Vytautas Bučinskas

**Affiliations:** 1 Institute of Science and Innovative Technology, Liepaja University, LV-3401 Liepaja, Latvia; 2 Department of Mechatronics, Robotics, and Digital Manufacturing, Vilnius Gediminas Technical University, LT-10105 Vilnius, Lithuania; jurate-jolanta.petroniene@vilniustech.lt (J.J.P.); vytautas.bucinskas@vilniustech.lt (V.B.)

**Keywords:** κ-carrageenan, force sensor, stretch sensor, iron (III) oxide, biodegradable

## Abstract

This article presents research on biodegradable stretch sensors produced using biological material. This sensor uses a piezoresistive effect to indicate stretch, which can be used for force measurement. In this work, an attempt was made to develop the composition of a sensitive material and to design a sensor. The biodegradable base was made from a κ-carrageenan compound mixed with Fe_2_O_3_ microparticles and glycerol. The influence of the weight fraction and iron oxide microparticles on the tensile strength and Young’s modulus was experimentally investigated. Tensile test specimens consisted of 10–25% iron oxide microparticles of various sizes. The results showed that increasing the mass fraction of the reinforcement improved the Young’s modulus compared to the pure sample and decreased the elongation percentage. The GF of the developed films varies from 0.67 to 10.47 depending on composition. In this paper, it was shown that the incorporation of appropriate amounts of Fe_2_O_3_ microparticles into κ-carrageenan can achieve dramatic improvements in mechanical properties, resulting in elongation of up to 10%. The developed sensors were experimentally tested, and their sensitivity, stability, and range were determined. Finally, conclusions were drawn on the results obtained.

## 1. Introduction

Structural concepts and materials inspired by nature help improve people’s lives worldwide. By preserving ecosystems and solving environmental sustainability issues, man achieves harmony with nature and creates well-being in his life. Materials found naturally in nature can be inexpensive, renewable, and suitable for various applications. Materials of natural origin biodegrade more easily than artificial ones and have biocompatibility and biorecognition features. Some such materials are valued for their self-repair and response to stimuli and may pose particular challenges because of inhomogeneity. Although some materials have been used for a relatively long time in the food industry or regenerative medicine, their use in functional devices such as (bio)electronics, sensors, and optical systems, which can also be used for healthcare and biomonitoring, is gaining more and more attention. Materials derived from plant and animal sources are currently under intense investigation as prospective renewable and inexpensive resources for various applications.

Various human activities require the detection of stretch. Flexible stretch sensors can efficiently convert mechanical force into electrical signals. They, as usual, are applied in motion monitoring, life and health monitoring, human–computer interaction, and other fields. Soft sensors are based on elastomers, and hydrogels with conductive fillers work on controlled electrical conductivity under applied force. Natural-origin hydrogel conductors are considered sustainable bioelectronic materials for wearable sensors and implantable devices because of their good biocompatibility when in contact with living organisms. Ion-conductive hydrogels (ionogels) are attractive as thin-film transistors, solid electrolytes, dielectric elastomer transducers, and materials for implantable bioelectronics due to the good biomimetic mechanical properties of such types of polymers [1]. One field of application is respiration sensors and physiological signal sensors [2], where hydrogels have an important place.

However, conventional strain sensors usually suffer from poor stretchability and compatibility between conductive fillers and elastomers, so preparing strain sensors that exhibit good stretchability and biocompatibility remains a challenge. Furthermore, there is an increasing need to develop portable, flexible, and safe energy storage devices to ensure long-term uninterrupted operation of portable electronic devices. State-of-the-art strain sensors have enabled the detection of sensitive and accurate joint bending, facial expressions, and swallowing and have excellent application possibilities. Such sensors usually contain nanostructures [3].

The aim of this paper is to present the possibility of creating a material for a biodegradable stretch sensor and to define its electric and mechanical characteristics.

## 2. State of Art

### 2.1. Polysaccharides as a Biopolymers

Polysaccharides, substances able to form a gel, are one of the substances abundantly found in nature [4]. These materials can be used as passive substrates or supporting architectures and often work as functional elements either by themselves or as biocomposites. Such products usually have mechanical properties that are incompatible with skin tissues and, therefore, face processing difficulties. Hence, as science progresses, there is a need to carefully evaluate materials that did not have appropriate qualities for previous applications.

The greatest success in developing sensors based on biologically derived hydrogels was achieved by obtaining a biocompatible, robust, and reusable sensor. Longevity has not always been a positive feature in recent years, as the modern sensor user wants integrated sensors in clothing and additional accessories and expects to change sensors frequently without polluting the environment and creating a lot of non-recyclable waste [5]. Careful analysis of biological materials and increased understanding of how they work and how they can be used, a joint pursuit of the entire scientific community, allow us to make assumptions and apply environmentally friendly materials.

As reported by several authors, naturally occurring ionic hydrogel-based sensors exhibit high voltage/pressure response over a wide range, can be used for the multimodal sensing of human activity signals, and are suitable for successful utilization. Properly prepared hydrogels can be freely reconstructed into new flexible electronics and safely integrated into human skin or any other relevant surface with complex terrain and complex motion. Some hydrogels, especially the carrageenan we studied, can be chemically processed to produce the final result of a cross-linked biopolymer [4].

Traditional hydrogels contain a large amount of water in their network and are weak against external mechanical stimuli. Hydrogels are excellent adhesive compounds because their hydroxyl groups interact with the phosphate groups of the human skin cell membrane by hydrogen bonding [6]. Ion-conductive hydrogel sensors have wide applications in wearable, implantable devices. Other authors have studied the rapid recovery of carrageenan gel, and this mechanical behavior is the result of the ionic bonds of carrageenan which are broken primarily during loading, thereby connecting the main structure of the base, which, in turn, is responsible for maintaining the hydrogel structure [7].

### 2.2. Natural Carrageenans

Carrageenan is a natural plant-origin biopolymer that occurs as matrix material in numerous species of seaweed. Carrageenans are gel-forming polysaccharides. More than 15 basic types of carrageenans are described and identified by a Greek prefix [8].

**Figure 1:**
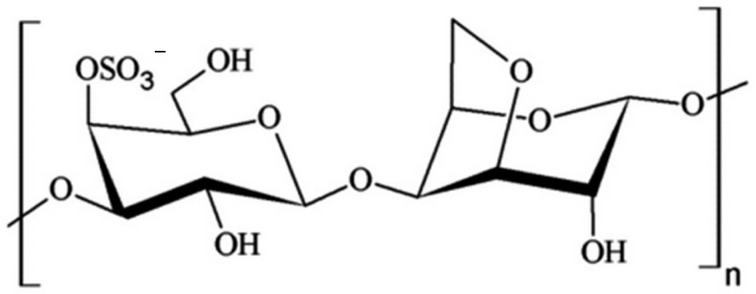
κ-Carrageenan molecule chemical structure.

There are three main types of carrageenan, but only iota and kappa are commonly used to form the gel or biopolymer (Figure 1).

Less-sulfated carrageenans have a marked ion specificity that is closely related to their gelling capacity [9]. Among the many seaweeds producing carrageenan, the furcellaria fastigiata-derived polysaccharide is named furcellaran, and this polysaccharide is distinct from carrageenan but is part of the carrageenan family. Furcellaria lumbricalis, like many other seaweeds, belongs to the phenotypic variation, and several morphological ecotypes are known [10]. Analysis of coastal benthic communities in the Baltic Sea shows a problem with seaweed reaching and contaminating the coast [11].

Carrageenan and furcellaran are extracted from the seaweed Furcellaria lumbricalis. They have some differences. Kappa carrageenan has one sulfate ester residue per three or four sugar residues, and furcellaran is a polysaccharide consisting of repeating beta and kapa carrageenan disaccharide units with lower sulfation levels, sulfated but negatively charged. Furcellaran is most famous as an antimicrobial and antioxidant material for biofilms. Furcellaran is more sensitive to heat drying when degradation is observed [12].

There are two known methods for extracting carrageenan from seaweed. The first is expensive because it begins with the extraction of carrageenan into an aqueous solution, and after the filtrate containing seaweed residues is removed, the carrageenan must be extracted from the solution. In the second method, seaweed is washed to remove solid impurities before being treated with alkali to extract carrageenan. In summary, when mentioning the process of extracting carrageenan from seaweed, we should mention the most relevant step: coagulation of the purified carrageenan liquid with isopropanol to obtain fibrous carrageenan coagulation [13]. Carrageenan gelation in the presence of cations in solution has been described as a conformational transition from random coils to helical structures that aggregate with decreasing temperature [14].

### 2.3. Natural Biopolymers in Industry and Sensor Manufacturing

Commercial carrageenans are applied as emulsifiers, stabilizers, and gelling agents in the food industry. Carrageenan, in addition to all the other mentioned fields of application, is considered a bioactive polysaccharide with disease-modifying and microbiota-modulating activities.

Such a solution when creating a biopolymer based on a natural hydrogel has its own problems because of the chaotic nature of the cross-linking of molecules. The mechanical behavior of such products is more difficult to predict. Still, considering the properties of natural materials, this disadvantage that appears at first glance can be turned into an advantage created by nature during thousands of years of improvement. Polysaccharides are the best of all types of natural polymers. Biopolymer-based hydrogels have weak mechanical properties because of their non-crystalline structure and cross-linking level.

Minimizing the distance between biopolymer fragments and increasing the number of cross-linkers are only a few ways to solve the mentioned problem [15]. Hydrogels are at the forefront of sensor development [16]. Shape-memory hydrogels in flexible strain sensor manufacturing look very attractive. They are attractive because of their good fit to curved surfaces and excellent conductivity. Gel polymers are widely used in industry because of their flexibility, self-healing, and other attractive properties.

Among the natural biopolymers that are currently actively applied in force sensor production, carrageenan has a wide range of applications. Wearable devices designed and assembled on hydrogels can be used to continuously and precisely detect whole body movement signals and collect electrophysiological signals such as electromyogram and electrocardiogram signals [17]. Typically, human skin deforms by up to 75% [18]. Therefore, many researchers working in the field have considered hydrogels very suitable candidates for skin-mounted motion sensors [19].

As a functional material in tissue engineering, drug delivery, and wound dressing, κ-carrageenan has certain advantages due to its immunostimulatory and antitumor activities. Therefore, there is an increasing need to develop strong and stretchy carrageenan hydrogels, especially the pure κ-carrageenan hydrogel, in order to avoid the side effects of hybrids with materials that are not suitable for contact with living tissues [20,21]. Carrageenan combinations with nanomaterials are increasingly used. However, using cheap, simple additives to develop new structures is an attractive research direction, primarily because nanoparticles that do not break down easily can pollute nature. Sensor collection and recycling must no longer be controlled, as irresponsible activities can spread in nature [22].

Ultrapure carrageenan is not always necessary to achieve the stated goals. In particular, semi-processed/semi-refined carrageenan is widely produced in various industries, including use as an additive in pet food and dairy products [23]. Carrageenan is also known to have a high molecular weight with an average molecular mass of about 1000 kDa or 106 g per mole [23]. Kappa-carrageenan has a linear arrangement of monomers, namely D-galactose-4-sulfate and 3,6-anhydro-D-galactose. The half-sulfate moieties in the carrageenan structure make it a strong anionic biopolymer [24].

The κ-carrageenan-based hydrogel can fragment under mechanical load. Various solutions have been sought to eliminate hydrogel disintegration problems. Great success was achieved by an improved hydrogel without the use of a second synthetic network. The sodium hydroxide with urea solution allowed for the regulation of the biopolymer chain from a random coil to a stiff chain structure, maintaining stress. When the hydrogel was added to KCl solution, more segments were incorporated, leading to the anisotropy of the material. Hydrogel modified in this way can recover and is suitable for wearable devices [20].

The deformable flexible structure must be adapted when conductive hydrogels are used in flexible strain sensors. Some authors have reported that a sandwich-like structure is usually adopted to prevent accidental damage to the gel sensor [25]. The development of flexible polymer films focuses on technologies that produce the required product using the simplest possible methods, such as the one-pot polymerization method [25]. The complex cross-linked structure containing the κ-carrageenan network and the structure developed from polyacrylamide with Fe^3+^ ions is reported to be a conductive ion pressure and strain sensor [26].

The shape memory hydrogel based on Fe_3_O_4_ nanoparticles, carrageenan, and polyacrylamide works as a self-adhesive electrically conductive material without additional ions. It is a promising double-network hydrogel actuator and strain sensor compound [6].

### 2.4. Natural Biopolymers in Combination with Plasticizers

Plasticizers are another important material to mention in the development of biopolymers suitable for force sensors. Typically, plasticizers are low-molecular weight, non-volatile compounds that are added to polymers to reduce brittleness, provide flow and flexibility, and increase film stiffness and strength. The most common disadvantages caused by plasticizers are that they reduce the intermolecular forces along the polymer chains; thus, they increase the permeability of the film to oxygen, moisture, aroma, oils, etc. [13]. Glycerol (25%) as a plasticizer with sorbitol was mentioned in the production of κ-carrageenan films, where the film showed the lowest tensile strength (TS) of 40.3 MPa and elongation at break (EAB) values (1.77%) compared to plasticized films with glycerol and sorbitol plasticized films [27]. Electrical conductivity was observed in κ-carrageenan with poly-acrylamide-co-acrylic acid without additional ions [6]. Carrageenan with polyacrylamide, glycerol, and ethylene glycol works as an excellent resistive-type sensor [28] with a wide range of relative humidity detection (4 to 90%) and good stability and linearity. Carrageenan is a versatile natural substance; it is indicated that carrageenan layers act as a capacitive moisture sensor, respiration sensor, resistive force sensor, etc. [2].

### 2.5. Natural Biopolymers in Modification and Combination with Other Polymers and Conductive Nanomaterials

Carrageenan mixed with other materials to form a polymer chain yields material suitable for force sensors. κ-carrageenan covalently cross-linked with polyacrylamide GF = 0.63 and 100% deformation works as a recoverable stretchable material [29]. Polyacrylamide and carrageenan with reduced graphene oxide (rGO) functioning as a dual network coated on the surface showed GF = 90.5 and a strain of 400%. The most interesting solution depicted in this work is to give these materials non-drying and antifreeze properties by replacing water molecules with 1,2-propanediol. The product did not freeze at −60 °C [30]. Some products are recovering/regenerating if hydrogel is accomplished by applying an external effect, such as heating the product to a suitable temperature. For example, the carrageenan hydrogel with Fe_3_O_4_ nanoparticles modified with acrylamide amine was successfully recovered by heating to 60 °C [6]. This hydrogel has a long lifetime as a result of its shape-memory behavior.

Conventionally κ-carrageenan (κCG)-based hydrogel can easily rupture under mechanical stress; therefore, developed carrageenan-based hydrogels are reinforced with additional solutions and fillers. A sufficiently strong hydrogel made from carrageenan was proposed without the use of a second synthetic network. To this end, polymer chains were adjusted from a random coil to a rigid chain conformation in the NaOH/urea solvent system through a freeze–thaw process [20]. The authors reported that the prepared hydrogel with a homogeneous structure could exhibit a higher elongation of 42.1 to 156%, recorded a rapid self-strengthening behavior, and obtained a more stretchable hydrogel (break strain up to 396%), stronger hydrogel (stress ~0.55 MPa), and tough (∼1.52 MJ m^−3^) κ-carrageenan hydrogel. Compared to the traditional material, break stress and hardness increase by 8.5 and 11.5 times, respectively.

Furthermore, this κ-carrageenan hydrogel displays good recovery and shape memory behavior under moderate deformation [20]. Carrageenan hydrogel-based sensors can be sensitive enough to detect various movements underwater, even monitoring pulses for healthcare in liquid environments. This type of hydrogel, with these comprehensive mechanical and sensing properties, has great potential in the fields of artificial intelligence and electric skins and continues to be used in aquatic environments [20].

There are also unique solutions that use the intrinsic fracture behavior characteristic of gels to realize self-growing materials that undergo bond rupture-initiated mechanical reactions, which would be applied to various gels and multi-network gels supplied with radically polymerizable monomers. Gels respond to repeated mechanical stimuli by increasing their polymer content and size and gaining more strength, similar to skeletal muscle growth induced by normal physical exercise. They applied programmed mechanical stimuli to DN gels fed the corresponding functional monomers [31].

The hydrogel produced by polymerizing acrylic acid, acrylamide, and N,N-dimethylacrylamide and cross-linked with zirconium hydroxide exhibited high mechanical strength [32]. One of the most practical ways to improve and modify the mechanical properties of polymers is by adding various organic, inorganic, and mineral particles. Adding even small amounts of fillers and forming polymer composites can significantly change their mechanical properties, such as Young’s modulus and tensile strength. Iron oxide, hematite (α-Fe_2_O_3_), is the most stable iron oxide and is known as a semiconductor, and this property of the material is very important scientifically and technologically [33,34]. Iron oxides are the most common iron compounds [35,36,37,38,39]. At least six nonhydrated crystalline iron oxides that exist in both bulk and nanoscale forms and are common in nature have been described. There are data that iron (III) oxide, being a neutral compound, can act as an n-type semiconductor. Rust, in general, is a poor conductor of electricity because it is an ionic compound in which ions cannot move freely [40].

Table 1 represents some GF elongation and conductivity values of sensors made using carrageenan and other compounds that polymerize with carrageenan. Most of the hydrogel yields a weak GF parameter. The GF value can be improved by enriching the hydrogel with nanocomposites, especially carbon nanostructures, and by complex final polymerization of carrageenan products.

The main goal of this work was to reevaluate the suitability of carrageenan as a biopolymer for use in force sensor manufacturing, guided by modern knowledge and modern tools and research methods and to identify a film sensitive to applied force and constructed without polymers and nanomaterials currently used in producing force sensors, with minimal composition. With this work, we offer a cheap and sustainable biodegradable product that is easy to create from natural resources. This carrageenan-based force sensor film with additives of natural origin is nontoxic to humans and nature.

## 3. Materials and Methods

### 3.1. Experiment Methodology

Experimental research was dedicated to developing a cheap and sustainable stretching sensor taking into account today’s sustainability ideas. Films containing different amounts of selected material were prepared. The prepared film was mounted onto a simple plastic structure with metal foil serving as connecting wires and was investigated using devices to measure the deformation under applied force.

### 3.2. Equipment

**Figure 2:**
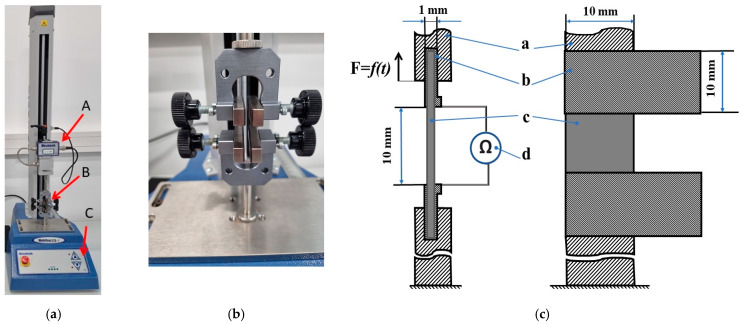
Mechanical stand for strain sensor testing with samples: (**a**) view of the mechanical testbench, where A—force sensor, B—clamps, C—manual control panel; (**b**) sample fixing clamps; (**c**) schematic representation of experiment: a—clamps, b—electrodes, c—carrageenan film, d—multimeter.

Tensile mechanical tests were performed using the computer-controlled tension–compression test system (Mecmesim Limited, Slinfold, West Sussex, UK, maximum load: 2500 N; maximum sample diameter: 134 mm; load sensor measurement error: ± 0.1%; speed range: 1 to 1000 mm/min) The testing machine was controlled by the software Emperor (Mecmesin Limited, Slinfold, UK) (Figure 2). Emperor force testing software takes full control of the test machine motor and collects data from the internal position sensor and force sensor. Figure 2a represents the structure of the mechanical test machine; its main parts are (A) a force sensor, (B) clamps for sample mounting, and (C) a manual control panel for adjusting the distance between clamps while mounting the samples. Figure 2c provides a structural scheme of the sensor prototype, consisting of the carrageenan-based polymer film installed between aluminum electrodes and compressed in the clamps of the tension machine. The deformable volume of the sensitive material between the electrodes is 10 × 10 × 1 mm. The stretching force was measured using the 1000 N load cell connected to the tension machine and attached to the upper clamp, as seen in Figure 2b. Resistance measurement was performed using the UNI-T UT55 Multimeter by connecting the electrodes to the opposite edges of the specimen at the same time.

The prepared samples were mounted on holders, and an applied tension test was performed at a 1 mm/min speed with a loading accuracy of 0.001 N. The test was carried out using a minimum of 3 identical samples produced individually under the same conditions. During the stretching tests, the samples were isolated from environmental factors (constant temperature and humidity) using additional very thin protective films, not affecting the mechanical characteristics of the tested polymer. After load experiments, some film samples were left in room conditions to assess their quality after five months.

The samples were investigated under load until the disintegration limit (Figure 5).

### 3.3. Definition of Sensor Parameters

One of the most important parameters for piezoresistive sensors is the gauge factor (*GF*). Its calculation is carried out as usual using the formula where resistance *R*_0_ is the initial resistance of the sample, *L*_0_ is the initial length of the sample, and *R* (Ω) and *L* (m) are the resistance and the length of the sample from the point of stretching, respectively.

(1) $$GF=\frac{\frac{R-R_{0}}{R_{0}}}{\frac{L-L_{0}}{L_{0}}}$$

The gauge factor (*GF*), the sensibility of a sensor, was obtained by linearly fitting the change curve of relative resistance.

The sensitivity was calculated:

(2) $$S=\frac{R-R_{0}}{F}$$

where *S* is the sample sensitivity, *R* is the maximum sensor resistance measured in (MΩ), *R*_0_ is the initial sensor resistance of the sensor in (MΩ), and *F* is the applied tension force in N.

Electrodes that connect the sensitive material of the sensor have a key influence on the sensor’s performance [43]. In this research, the sensor was identical in size, material and fixing method; therefore, the electrode on the resting procedure was outside the scope of this research.

The conductivity of sensor samples is defined as follows:

(3) $$\sigma =\frac{1}{R}\cdot \frac{L}{s};$$

where *σ* [S m^–1^] is the conductivity, *s* [m^2^] is the cross-sectional area, and *L* [m] is the distance between the two electrodes.

Maxwell’s model describes a stress that decreases exponentially with time. This property applies to most polymers. The disadvantage of this model is that it does not accurately predict potential creep. Thus, Maxwell’s model for creep or permanent stress conditions states that stress will increase linearly with time [44].

### 3.4. Development of a Sensor Prototype

As the main material for the force sensor, we selected the carrageenan polysaccharide without additional polymers to enhance the structure of the film. To realize this idea, we made the film using only the main materials: polysaccharide biopolymer, simple plasticizer, and a compound sensitive to applied force.

#### 3.4.1. Extracting of Carrageenan Biopolymer from Seaweed

Carrageenan was prepared from furcellaria lumbricalis seaweed in the scientific lab of Liepaja University, Latvia, according to laboratory protocols [45,46]. Most carrageenans are precipitated by the addition of alcohol, usually isopropanol, yielding coagulum, which is fibrous in moisture. This procedure is applicable to potassium-sensitive carrageenans or κ-family carrageenans [47]. Kappa-carrageenan was partially removed from filtered seaweed moieties. Thus, the jelly extracted from seaweed was heated to a temperature of 70 °C in a water bath and suspended in 2-propanol in a vessel equipped with a reflux cooler while stirring the solution slowly. After biopolymer formation was noticed, the product was filtered through a sieve with a one-millimeter density. Before all of the subsequent steps, the prepared carrageenan mass was stored at a temperature of 9 °C.

#### 3.4.2. Sample for Stretch Sensor Preparation

All solutions were prepared using deionized water. All chemicals (isopropanol, iron oxide (III), and glycerol) were purchased from Sigma-Aldrich. All aid materials, such as aluminum foil, transparent plastic plates, etc., were obtained according to the description of the laboratories’ internal procedures.

**Figure 3:**
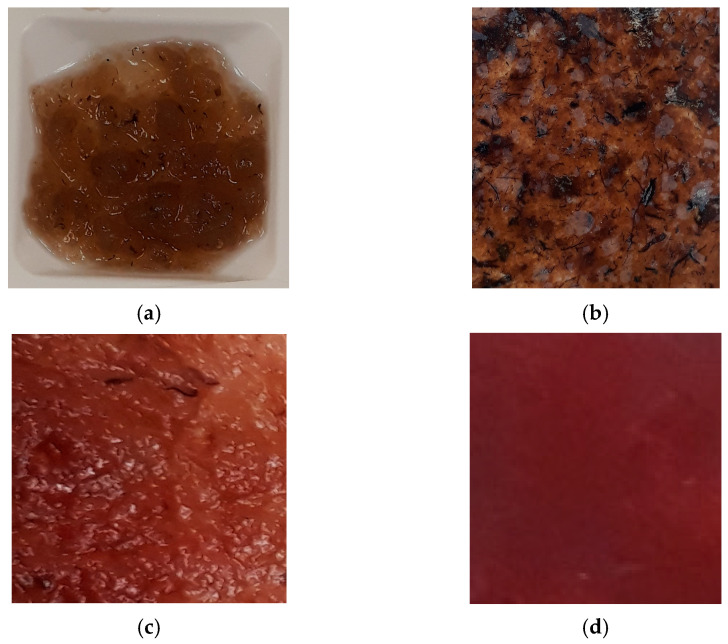
Steps of sample preparation from carrageenan precipitates to film. (**a**) The precipitates of carrageenan. (**b**) Dried pure carrageenan film. (**c**) Precipitates of κ-carrageenan with iron (III) oxide. (**d**) Dried film of carrageenan with iron (III) oxide.

As already mentioned, the produced carrageenan sediments were selected for the next steps of film preparation. Figure 3a–d represents the steps of this work during the preparation of the carrageenan film. To evaluate the physical properties of pure carrageenan, we performed the same measurements as with the subsequently prepared force sensor film.

As shown in Figure 2a,b, incompletely purified carrageenan was selected from residues from seaweed plants for this research. As we know, the purification of this substance, like any other, requires additional resources, and this process is not sustainable. The precipitates prepared mixed with iron oxide (III) and glycerol (Figure 3c) were left to dry under room conditions. Properly dried film is flexible, soft, and non-greasy and recovers after compression (Figure 3d). According to experiments, this quality is maintained for at least five months, even without any insulation or laminating of the specimen.

Figure 3 represents the complexity of this naturally derived polysaccharide transformation from seaweed to a soft electroconductive film. A pure carrageenan film without main ingredients was applied as a material for the “baseline” experiments in order to understand how this type of carrageenan works as a tension experiment sample. In Figure 3c, the prepared mixture looks like a rough relief, but after drying (Figure 3d), the film has a sufficiently uniform thickness.

**Figure 4:**
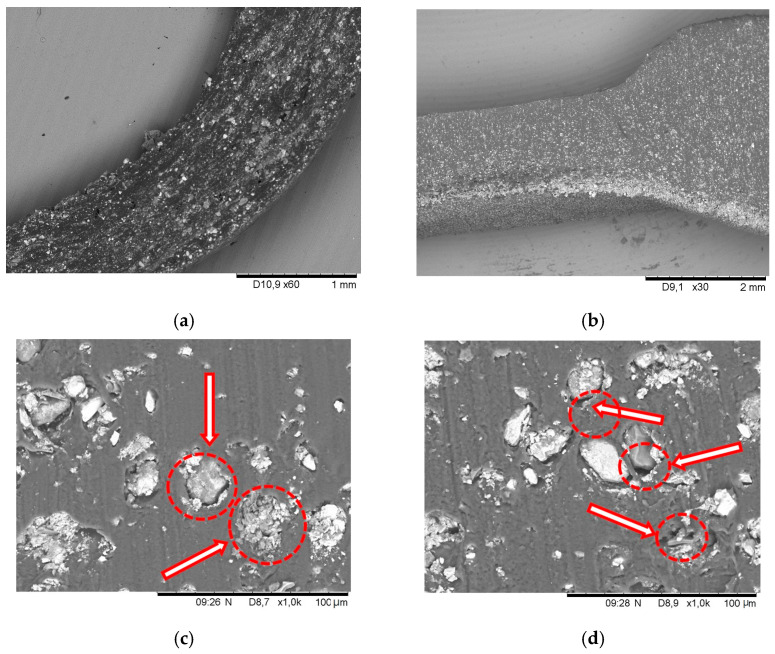
Investigation of the κ-carrageenan biopolymer film with Fe_2_O_3_ structures by SEM. (**a**) Micrograph of carrageenan biopolymer film cross-section with iron oxide Fe_2_O_3_ particles. Size = 1280 × 1100; DPI = 182.65; Conditions: V_acc_ = 15.0 kV; Mag- = ×60; WD = 12.40 mm. Pixel size = 231.771. (**b**) Micrograph of carrageenan biopolymer film with iron oxide Fe_2_O_3_ particles. Size = 1280 × 1100; DPI = 182.65; Conditions: V_acc_ = 15.0 kV; Mag- = ×30; WD = 10.60 mm. Pixel size = 4635.42. (**c**) Size = 1280 × 1100; DPI = 182.65; Conditions: V_acc_ = 15.0 kV; Mag- = ×1000; WD = 10.20 mm. Pixel size = 139.06; (**d**) Size = 1280 × 1100; DPI = 182.65; Conditions: V_acc_ = 15.0 kV; Mag- = ×1000; WD = 10.40 mm. Pixel size = 139.06.

As mentioned above, the prepared carrageenan was mixed with Fe_2_O_3_ and glycerol in different proportions. The visual representation of the sample structure is Figure 4, presenting micrographs performed by SEM. As we can see from the micrographs at the lowest magnification (Figure 4b), the iron oxide particles were uniformly distributed over the entire surface of the film. Some irregularities were observed in the cross section of the film (Figure 4a), where we can recognize irregularities in the film’s iron (III) oxide particle distribution. These results are attributed to the viscosity during mixing and the force of gravity.

As seen in the micrographs, the structure of the films is complicated. Unseen iron (III) oxide was selected as the industrial pigment for the manufacturing of the film. This selection resulted in iron (III) oxide particles of various sizes, and some of them are also broken into smaller particles, as seen from the micrograph in Figure 4c. We observed some air gaps around most iron oxide particles (Figure 4d). These air gaps affect the initial contact of iron (III) oxide particles with conductive biopolymer film and determine the value of the initial measured resistance. It is obvious that this contact changes when the product is stretched. It is very likely that the resistance will decrease just before the rupture of such a film as a result of the increased contact between the iron (III) oxide particles. We observed such phenomena but did not include these data for the calculations and represented the most linear parts of the applied force curves that met the requirements for force sensors.

Experiments were performed at ambient temperature, standard controlled in laboratory air pressure and humidity. Successive tensile tests were performed until the specimen broke. Once a successful, tear-resistant product formulation was found, subsequent tests were performed immediately without delay. Rectangular hydrogels with dimensions of 20 ± 1 mm (L) × 10 ± 1 mm (d) × 1 ± 0.1 mm (w) were used for the measurements, and the stretching rate was set at 1 mm/min. Unless otherwise indicated, all data are presented as the mean ± SD of at least three independent experiments.

Following preliminary tests, a specific amount of iron oxide was selected to produce carrageenan films. The amount of glycerol used to produce carrageenan films was selected based on publications and additional research. The mixed components were evenly spread on a plastic Petri dish and dried at room temperature. After visual evaluation of film quality, it was cut into pieces of selected size. Carrageenan film strips were placed with aluminum strips in the holders as contact electrodes. The electrodes were made of aluminum due to its good compatibility with carrageenan-based biopolymers and well-analyzed contact interaction. Due to the structure of the biopolymer and the electrochemically active sulfate groups present in the biopolymer (Figure 1) and the natural acidic media nature of carrageenan, this polysaccharide contains water molecules and performs connection to the aluminum surface using electrons of the outer layer.

## 4. Results

The results of the experiments show that the carrageenan biopolymer acts as a film-supporting structure. Iron oxide pigment has already been successfully applied as a force sensor material by our research group in sensor development [48]. The load applied to the sample stretches the carrageenan structure and causes the iron (III) oxide powder particles to move within the film structure. The created load causes a rearrangement of the charge transport path, or, in other words, the material’s conductivity, and is recorded as a change in measured electrical resistance. As already stated in previous work of this scientific group, the electrical conduction mechanism within the powder can be attributed to percolation, quantum tunneling, and the semiconductor properties of Fe_2_O_3,_ depending on the distance between the particles and their physical interaction with them. After reviewing the most outstanding results, we represented data from a few examples whose mechanical test data represent the most suitable composition of the film.

**Figure 5:**
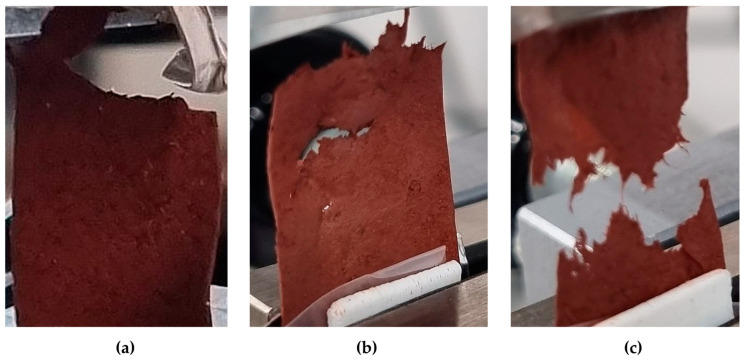
View of films damaged by the applied force. (**a**) Broken carrageenan film, the composition with 0.5 mL of glycerol and 3 g of iron (III) oxide; (**b**) sample with 0.05 g of iron (III) oxide; (**c**) cracked specimen of carrageenan film, the composition with 3 mL of glycerol.

The tensile experiment was carried out until the sample completely ruptured. As shown in Figure 5a, the sample tear differs in comparison to Figure 5b and depends on the amount of iron oxide and glycerol. The sample with a higher amount of iron (III) oxide tears in a straight line. Its splinters are parallel to the line of mounting into the holders. The film containing a higher concentration of glycerol is less rough, softer, and weaker in tensile strength tests, and its tear edge has a fibrous structure. It can be assumed that the samples containing more glycerol rupture before the biopolymer “threads”.

**Figure 6:**
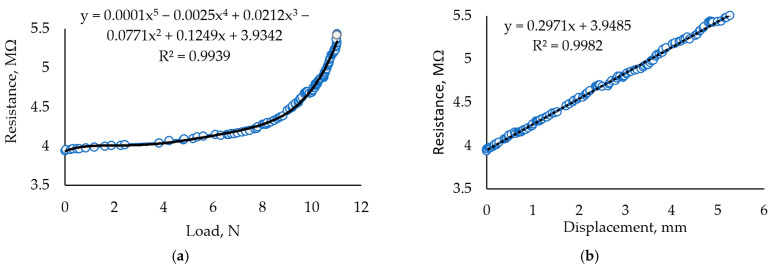
Investigation of samples with 30 mL Crgn + 0.5 mL Glc + 1 g Fe_2_O_3_. (**a**) Dependence of resistance under controlled force. (**b**) Dependence of resistance on the registered displacement of the sample during the mechanical load.

Thus, the composition of the carrageenan film within the investigated limits is best when the composition is 30 mL of carrageenan with 0.5 mL of glycerol (Glc) and 1 g of Fe_2_O_3_ dried under ambient conditions. The results of the mechanical load of the sample containing 0.5 mL of glycerol or a lower concentration of this plasticizer and 1 g of Fe_2_O_3_ are represented below (Figure 6a,b).

According to the calculations, the calculated conductivity of a sample using Formula (3) at the initial moment of the experiment was 0.092 mS, but the conductivity at the film rupture was 0.0012 mS. This can be attributed to the phenomenon of the reduction of distance between active groups in the biopolymer during applied load. As already mentioned, this is determined by the change in the distance between the iron oxide particles and the change in the diameter or charge-transferring width of the polymer sample.

Analogous information on other samples is represented in Table 2. The figure (Figure 6a,b) contains data from a complete applied force experiment that covers the sensor’s operating zone, as well as data that are not suitable for the sensor but which show the changes of resistance occurring during the destruction of the carrageenan film (Figure 5a). In the following sample composition, the amount of glycerol and iron (III) was increased to 3 mL glycerol and 3 g Fe_2_O_3_ in the same amount of carrageenan. The applied load experiment shows that the linear dependence of the registered resistance on the applied force is within 0.7 mm of displacement. Although glycerol gives the sample some softness and elasticity, its large amount weakens the film.

The sample parameters after aging under natural room conditions without sealing result in a decrease in the calculated parameters, so sealing of the film is necessary. As we can see from several examples, GF is influenced by many factors, including the aging of the product.

**Figure 7:**
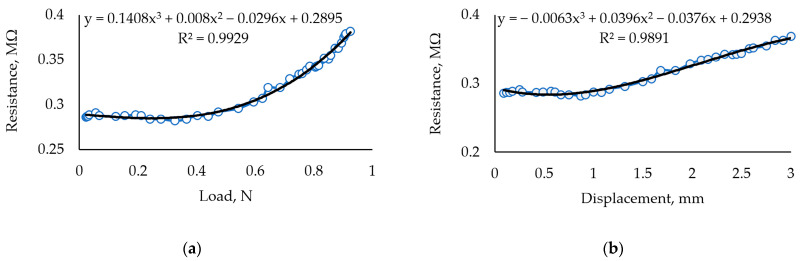
Investigation results of the sample containing the initial material proportions of 30 mL Crg + 0.5 mL Glc + 1 g Fe_2_O_3_ after 5 months of aging. (**a**) Dependence of resistance on the force applied to the sample. (**b**) The dependence of the measured resistance on displacement resulted from the applied load.

Figure 7 presents characteristics analogous to those presented in Figure 6 but defined after five months of aging. To clarify, Figure 6a,b and Figure 7a,b represent experimental data of the sample used for the experiment as soon as we prepared the sample and other parts investigated after five months of aging. Comparing the mentioned figures, it can be concluded that the R^2^ of the fresh one-sample approximation curve corresponds to the polynomial 5^th^ order, and that of the aged sample corresponds to the polynomial 3^rd^ order. Such differences in approximation curves probably reflect changes in the material structure during product aging. By applying analysis of other authors working with biogels, we can claim that hydrogel biodegradation is accelerated when pH is decreasing, so slow cross-linking can influence biodegradation [49]. However, analyzing the kinetics of this reaction in more detail is not appropriate because of the unevenness of the biopolymer structure. However, this assessment is not useful for hydrogels applied in the field of mechanical sensors.

Significant changes in material mechanical and electrical characteristics, such as decreased elasticity and overall resistance, are seen. This can be explained by the variation of the humidity in the material and the local relocation of conductive particles. This assumption is supported by the resistance and displacement dependency (Figure 7b), showing a more chaotic process than the newly prepared material.

**Figure 8:**
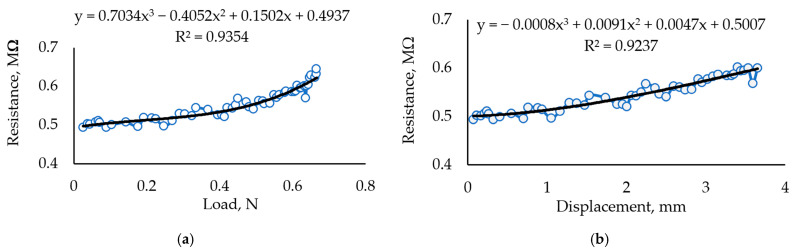
Investigation of sample with 0.5 mL glycerol and 0.05 g Fe_2_O_3_ in 3 g of carrageenan. (**a**) Dependence of resistance on the force applied to the sample with 30 mL Crg + 0.5 mL Glc + 0.05 g Fe_2_O_3_. (**b**) Dependence of resistance under displacement.

Figure 8 shows the characteristics of samples containing 0.05 g Fe_2_O_3_. This composition results in lower elasticity and stability. Chaotic resistance variations in general trends are noticeable and reach up to 100 kΩ. Such behavior signals that the amount of Fe_2_O_3_ is close to the critical value and that the pathways for electric current to travel through the media are very unstable.

**Figure 9:**
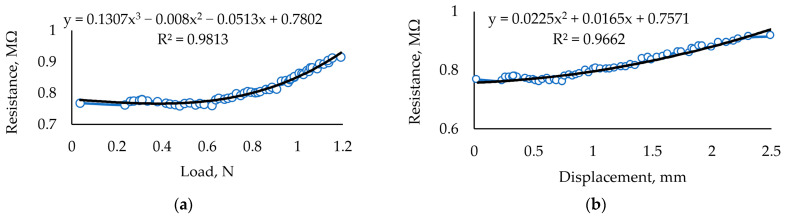
Investigation of the sample with 30 mL carrageenan + 0.5 mL glycerol + 0.05 g Fe_2_O_3_; sample aged 5 months. (**a**) Dependence of measured resistance on the applied load. (**b**) The dependence of measured resistance on displacement resulting from the applied load.

After 5 months of aging, this composition showed an improved response (Figure 9). Overall resistance increased due to a lowered humidity level, but the chaotic behavior disappeared. Elasticity decreased by about 25% compared to the 45% decrease in the first sample, showing that the concentration of Fe_2_O_3_ significantly impacts the characteristics of the biopolymer and its aging process when the material is not protected from the environment. The negative impact of aging is a decreased sensor sensitivity in the load range of up to 0.5 N.

**Figure 10:**
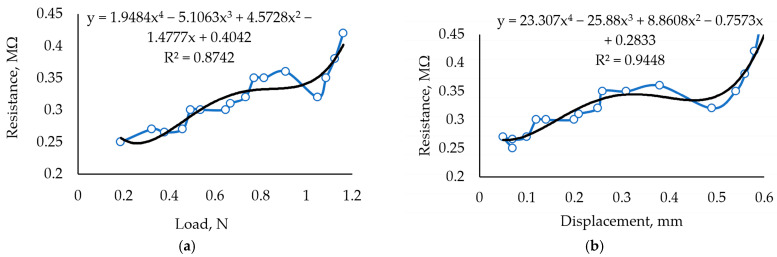
Investigation of a sample with 30 mL carrageenan + 3 mL glycerol+ 3 g Fe_2_O_3_. (**a**) Dependence of measured resistance on the applied load. (**b**) The dependence of measured resistance on displacement resulting from the applied load.

By comparing of results in Figure 8 and Figure 9, the R^2^ fits the experimental curve by using a polynomial of the third order and the aged sample shows the best results of fitting. These results correspond to the material properties when the cross-linking reaction of κ-carrageenan is a slow process, but the composition of this sample shows better results than the samples represented in Figure 6, Figure 7, and Figure 10. Thus, it is obvious that the components used for the sensitive material of the sensor are determined by the quality of the raw material, and the produced specimens reveal how the film quality deteriorates with an excess of glycerol or iron oxide.

An increased Fe_2_O_3_ concentration of up to 3 g resulted in lower film strength and unstable sensitivity (Figure 10), showing that a too-high Fe_2_O_3_ concentration interferes with the formation of a homogenous biopolymer structure required for good elasticity.

**Figure 11:**
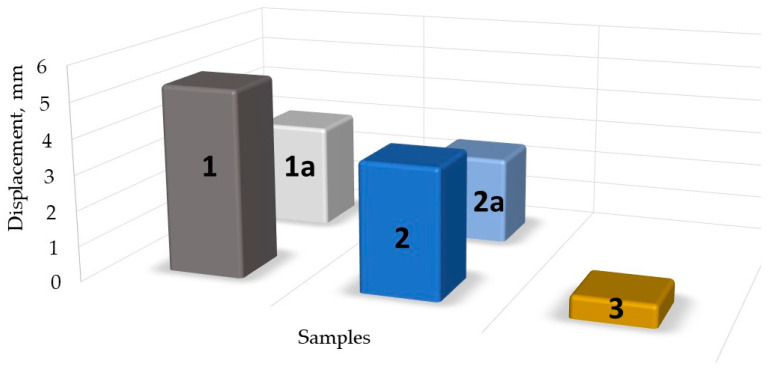
Visual representation of the displacement of samples; **1** and **1a**: 30 mL Crg + 0.5 mL Glc + 1g Fe_2_O_3_ and 30 crg + 3 mL Glc + 3 g Fe_2_O_3_; **2** and **2a**: 30 mL Crg + 0.5 mL Glc + 0.05 g Fe_2_O_3_; **3**: 30 mL Crg + 3 mL glycerol + 3 g Fe_2_O_3_.

Thus, evaluating the experimental data presented in Figure 6, Figure 7, Figure 8, Figure 9 and Figure 10, it can be seen that the film produced can be applied as a film for a force sensor, but some problems are posed for the repeatability of the developed product. Carrageenan biopolymer film enriched by glycerol plasticizer and iron (III) oxide is sensitive to the proportions of these few simple compounds and the aging process of the prepared composition. Figure 11 compares the maximum displacement (stretch) of all samples tested until they were broken. As seen from Figure 10, glycerol hinders the quality of carrageenan molecules’ binding.

As seen in Figure 11. the displacement varies with film composition. The highest elasticity that allowed for film elongation of almost 6 mm was achieved with a composition of 30 mL Crgn + 0.5 mL Glc + 1 g Fe_2_O_3_. The composition of 30 mL Crgn + 0.5 mL Glc + 0.05 g Fe_2_O_3_ achieved an elongation of almost 4 mm. Aged versions of both compositions resulted in an elongation of 3 and 2.5 mm, respectively. The composition containing a high concentration of glycerol and Fe_2_O_3_ (30 mL Crgn + 3 ml Glc + 3 g Fe_2_O_3_) resulted in a pore elasticity of only 0.6 mm. Nevertheless, it is still suitable for some indicative applications.

Considering the elongation of the sensor and its sensitivity, the best characteristics are demonstrated by the aged version of the composition with 30 mL Crg + 0.5 mL Glc + 0.05 g Fe_2_O_3_. However, although carrageenan cross-linking was confirmed two decades ago, the properties and combination of composite concentrations are still not straightforward, as can be seen from the experimental data. However, this result indicates that by operating with a composition made from these few materials, such a film’s electrical and mechanical properties could be adjusted according to individual needs.

## 5. Conclusions

The results show that carrageenan-based biopolymers complemented by glycerol and Fe_2_O_3_ are promising solutions for developing inexpensive adjustable stretch sensors following the latest trends in sustainability and biodegradation.

The aged version of the composition with 30 mL Crg + 0.5 mL Glc + 0.05 g Fe_2_O_3_ showed the most promising and stable results. This composition ensures 2.5 mm elongation, 0.225 MΩ/N sensitivity, and a force measurement range up to 1.15 N, showing that the composition of the material and its post-processing could significantly affect the overall performance. From the experimental results, the best composition is probably about 1.6% glycerol and about 3% Fe_2_O_3_ for carrageenan extracted from the weeds of the Baltic Sea.

Biodegradable carrageenan-based stretch and force sensors have promising perspectives in flexible and soft devices as a single sensor; they can be combined with various load cells as a sensitive force or torque sensor element. Piezoresistive films installed as membranes can be used as pressure sensors, and finally, there are potential stretching and bending wearable sensor applications. The designed sensors are limited by stretching values of 10% and are sensitive to environmental conditions. On the other hand, they are applicable as stickers that do not require ungluing and deposition due to biodegradation. This feature is very useful in packing applications for monitoring purposes.

The obtained results validated the initial hypothesis and proved the functionality of the proposed materials; nevertheless, there are a lot of questions for further research. First, it is necessary to solve sensor encapsulation issues. Sensor material should be protected from environmental impact. Second, it is necessary to perform detailed research on remaining characteristics, such as hysteresis, response time, sensitivity drift, etc. Third, the biodegradation process of the proposed material must be evaluated. Reliable electrical contact between the electrode and the film is also crucial in producing stretchable sensors.

## Figures and Tables

**Table 1:** Parameters of carrageenan-incorporating force sensors with different compositions.

Composition of Film	Gauge Factor	Elongation	Conductivity	Ref.
magnetic nanocomposite hydrogels: *N*, *N*-methylene bisacrylamide (MBA) + N, N, N′, N″, N″-pentamethyldiethylenetriamine bonded carrageenan	PAA/kCG—1.19 MPAA/kCG—1.41	8% 6%		[6]
PVA. CaCl_2_, yota-carrageenan,	1.33	20%		[19]
K-CG/P(Aam-co-AAC) Fe_3_ + hydrogel from κ-carrageenan + acrylamide (AAm), acrylic acid (AAc), and acryloyl chloride	0.78 to 2.8	1400%	1.15 Sm^−1^	[26]
κ-carrageenan + polyacrylamide (PAAm) double networks	0.63	1000%	n/a	[29]
κ-carrageenan (K + C) microgel (MG) composite hydrophobically cross-linked polyacrylamide (HPAAm) gels or K + C-MG/HPAAm gels	0.745	n/a	n/a	[31]
Hydrogel based films	0.6 to 11	100%	8.14 ± 0.50 Sm^−1^	[41]
rGO + polyacrylamide (PAM) + carrageenan dual network	4.6 to 90.5	n/a	n/a	[30]
acrylamide (AM) and N, N-dimethyl acrylamide (DMAA) (AMD gel)	1.0 to 3.1	1400%		[32]
polyacrylamide Li +/carrageenan	1.83	200% to 567.7%	1.9 Sm^−1^	[42]

**Table 2:** Parameters of carrageenan force sensors with different compositions.

Sample Info: Composition, (Figure Number)	Conductivity, mS	GF	Sensitivity, MΩ/N
30 mL Crg + 0.5 mL GLc + 1 g Fe_2_O_3_ (Figure 6a,b)	0.002 to 0.08,	1.43	0.134
30 mL Crg + 0.5 mL Glc + 1 g Fe_2_O_3_ aged for 5 months (Figure 7a,b)	0.104	0.67	0.104
30 mL Crg + 0.5 mL Glc + 0.05 g Fe_2_O_3_ (Figure 8a,b)	2.10 to 0.64	10.472	0.221
30 mL Crg + 0.5 mL Glc+ 0.05 g Fe_2_O_3_, aged for 5 months (Figure 9a,b)	2.6 to 2.1	3.12	0.225
30 mL Crg + 3 mL Glc + 3 g Fe_2_O_3_ (Figure 10a,b)	0.0012 to 0.092	4.16	0.123

## Data Availability

The data presented in this study are available on request from the corresponding author.
